# Do genetic variants of the Y chromosome affect mortality from COVID-19

**DOI:** 10.1177/14034948251333236

**Published:** 2025-04-14

**Authors:** Ole Bernt Lenning, Ronny Myhre, May Sissel Vadla, Roald Omdal, Begoña Martínez Jarreta, Ángel Gómez Moreno, Ignacio De Blas, Geir Sverre Braut

**Affiliations:** 1 Research Department, Stavanger University Hospital, Stavanger, Norway; 2 Norwegian Institute of Public Health, Division of Health Data and Digitalization, Department of Genetics and Bioinformatics (HDGB), Oslo, Norway; 3 University of Stavanger, Bergen, Norway; 4 Research Department, Stavanger University Hospital, Clinical Immunology Research Group, Stavanger, Norway; 5 Facultad de Medicina/Faculty of Medicine, Universidad de Zaragoza/University of Zaragoza, Zaragoza (Spain), Spain; 6 Dpto. of Hispanic Literature and Bibliography, Universidad Complutense de Madrid, Madrid, Spain; 7 Facultad of Veterinary Sciences, Instituto Universitario de Investigación Mixto, Agroalimentario de Aragón (IA2), Universidad de Zaragoza, Zaragoza, Spain; 8 Research Department, Stavanger University Hospital and Department of Social Science, Western Norway University of Applied Sciences, Stavanger, Norway

**Keywords:** COVID-19, Y chromosome, haplogroups, immunity, mortality, sex bias, pandemic, epigenetic, evolution

## Abstract

**Aims:**

During the early stages of the COVID-19 pandemic, significant differences in mortality patterns emerged based on sex and geographical regions. While we were studying on the heredity of variants of the Y chromosome, we observed that regional variations in mortality rates appeared to correlate with the geographical distribution of certain variants of the Y chromosome. This observation led us to propose that some genes on the Y chromosome, with an influence on immune responses, may represent a confounding factor in the observed geographical mortality differences.

**Methods:**

In this analysis, we investigate the potential associations between COVID-19 morbidity and disease-specific mortality and specific Y chromosome variants. The study is based on publicly available pandemic data validated by state authorities or presented in scientific literature documented in PubMed and Medline.

**Results:**

We find that Y chromosome haplogroups in different populations exhibit wave-like patterns corresponding with persistent global disparities in COVID-19-related mortality.

**Conclusions:**

**These findings warrant further research to uncover possible new pathophysiological mechanisms.**

## Introduction

Already in the early phase of the COVID-19-pandemic considerable differences in the disease burden could be observed, even between neighbouring countries. Four years into the pandemic, disparities regarding the severity and mortality of COVID-19 across different geographical regions still exist. Notably, men experience higher severity and death rates compared with women [1, 2]. While some of these differences can be attributed to variations in age distribution and life expectancy, socioeconomic conditions, regional preventive measures and different reporting routines, other factors remain unclear [3–5]. However, it is possible to identify traits of covariation between the disease burden and the distribution of certain genetic characteristics in different populations, in particular related to the Y chromosome, which so far remain unexplained [6–11].

This article aims to initiate an analysis of the role of biological sex and population genetics behind these disparities.

**Figure 1.**
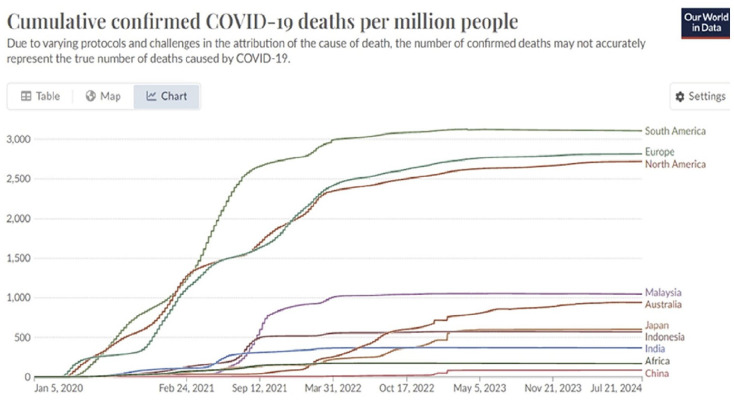
The impact of COVID-19 varied across countries, early and severely affecting Europe, North and South America, unfolding in unique timeframes and wave patterns. Data source: Our World in Data. WHO COVID-19 Dashboard [12].

Differences in national registration and reporting standards for COVID-19 cases and deaths complicate data comparisons. Europe and the Americas, representing a quarter of the world’s population, account for three-quarters of global COVID-19- reported deaths according to Our World in Data and World Health Organization (WHO) (Figure 1). Excess mortality reports at different times during the pandemic, in which cumulative excess mortality from the start of the pandemic of all causes compared with an extrapolation of historical data, can improve our level of knowledge of a pandemic mortality wave pattern [12].

Early global meta-studies highlighted a significant sex bias in COVID-19 morbidity and mortality, with men having nearly three times higher odds of needing intensive care unit treatment and a death rate 1.39 times higher than women, even after adjusting for comorbidities [1, 2].

Previous coronavirus outbreaks, such as the SARS-CoV-1 epidemic, showed similar sex biases, with men having a higher age-adjusted relative mortality risk ratio [14]. These disparities are more or less influenced by socioeconomic and environmental factors, in addition to biological factors such as hormones and differences in immune response between men and women [15–21]. Autoimmune diseases are on the whole more prevalent in women, and vaccine responses are stronger [22].

## Sex chromosomes and COVID-19

The X and Y chromosomes play a crucial role in determining biological sex and have garnered increased attention for their impact on sex-specific differences in disease susceptibility, severity, and outcomes, particularly in the context of COVID-19. The X chromosome with over 1000 genes holds some immune-related genes with relevance to COVID-19, such as the angiotensin-converting enzyme 2 (ACE2) virus receptor gene, toll-like receptor 7 (TLR7), and the androgen receptor gene (AR), which are known to appear differently in men and women through hormonal influence, genetic and epigenetic factors [15, 17, 20, 23–26].

In contrast, the Y chromosome, traditionally viewed primarily as a sex-defining chromosome, has emerged in recent years as an interesting player in influencing immune and inflammatory responses in men [16, 27]. It contains approximately 50 genes including a few immune-related genes [15].

## Y chromosomal haplogroups correlates with COVID-19 lethality

Haplogroups are alleles that are inherited together and thus reflect ancestral migration. Y chromosome haplogroups are named A–R used to define genetic populations following the same patrilineal line [28]. Polymorphisms of these genes contribute to variations in immune responses, with specific Y chromosome haplogroups potentially linked to disease progression. For instance, studies have associated Y chromosome haplogroup I with the progression of HIV to AIDS and genetic variations on the Y chromosome with susceptibility to influenza A virus [29, 30].

An intriguing avenue of exploration is whether these Y chromosomal haplogroups exhibit immunological variations that result in geographically distinct disease burdens over time in the face of a mutating virus. This raises questions about the potential impact of Y chromosomal genetic diversity on disease outcomes and responses to viral infections and COVID-19. For example, the Y R1b haplogroup has been associated with increased COVID-19-related deaths in European countries during 2020 [6–8].

Delanghe et al. documented a correlation between Y haplogroup Y-R1b-S116 frequency and COVID-19 mortality in European populations, while a recent study in northern Spain demonstrated a significantly higher risk of severe illness in the Y-I and Y-R1b haplogroups [9, 31, 32]. A European whole genome association study (GWAS) indicated a possibility for an association with haplogroup R1b1a2a1 (U106) in both the Spanish and Italian populations, and emphasises the need for large-scale studies [11].

## Geographical adaptations and COVID-19

Geographical adaptations, including genetic variations such as single nucleotide polymorphisms (SNPs) and epigenetic mechanisms, have played a pivotal role in human evolution and may influence susceptibility to COVID-19. For instance, the COVID-19, ACE receptor is particularly androgen sensitive, highlighting the possibility for Y chromosome genes that modulate androgen receptors across different haplogroups [26, 33–35]. Exploring the potential impact of a mutating virus on varying Y chromosomal haplogroups in different geographical regions, and their susceptibility to disease severity over time, is therefore an interesting area of study.

**Figure 2.**
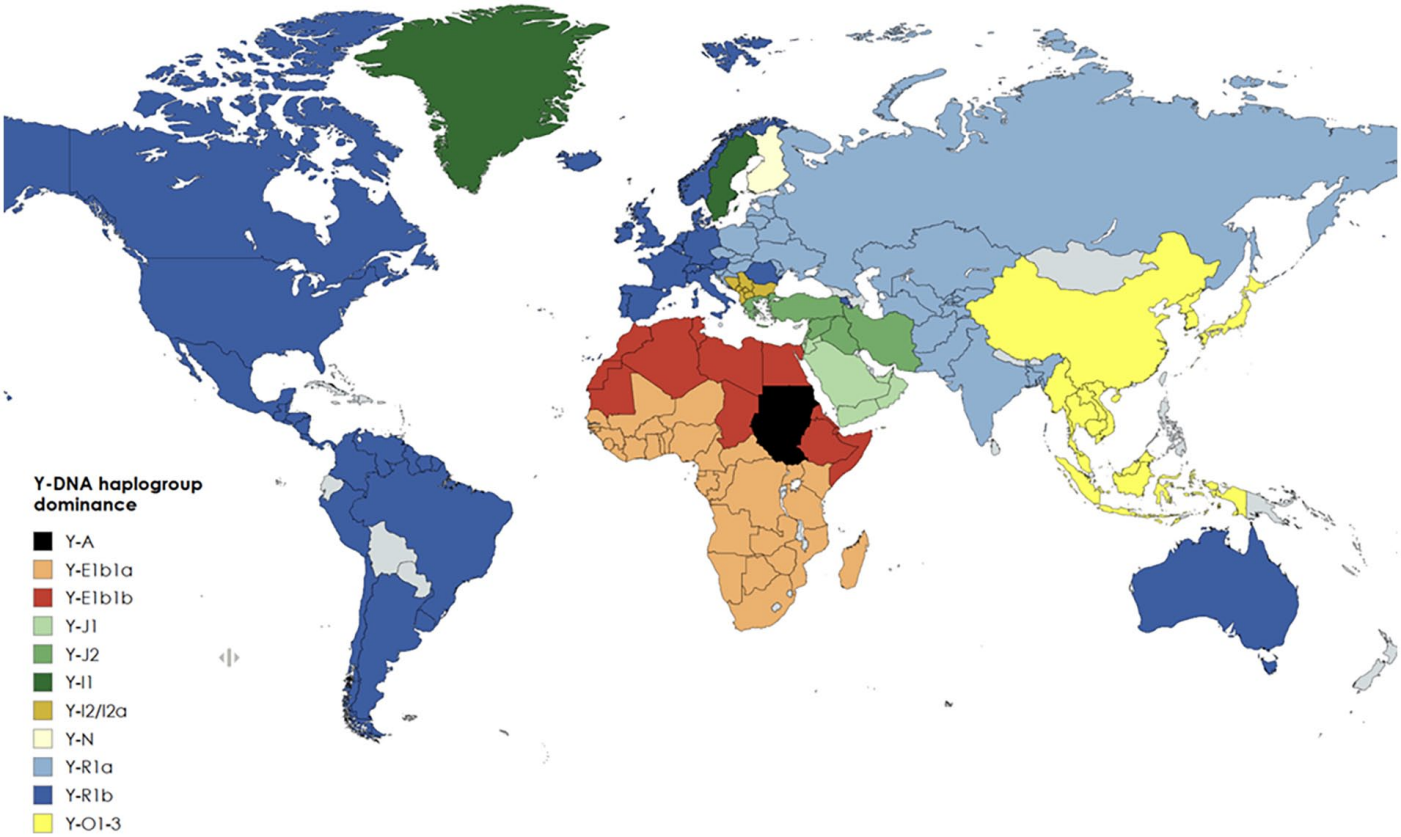
Current global Y haplogroup distribution by dominance in each country [36–47]. Created with mapchart.net.

The Y-R1b population (Figure 2 (dark blue)) representing the Caucasians is defined by a mutation that emerged near the Caucasus and the Caspian Sea after the last ice age and migrated west in the Bronze Age, becoming dominant in Western Europe [36]. On migration from Europe (Columbian Exchange), Y-R1b later became dominant in North America and in South America, where the indigenous population represented by Y-Q was greatly reduced [45]. According to public health reports, these Y-R1b dominated areas seemed to be severely affected with high COVID-19 mortality in the first wave of the pandemic [13]. In northern Italy, Y-R1b occurs very frequently, with, for example, well over 80% in the hard-hit Bergamo area. The second wave affected all Eastern Europe and the Balkans very hard, with reported high COVID-19 mortality rates where the Slavic population is dominated by Y-R1a (Figure 2 (light blue)) and the Balkans by Y-I2 (Figure 2 (brown)). India, which was affected severely remarkably late and in the third pandemic wave, has a composite Y haplogroup picture, with Y-R1a dominating. The whole of South East Asia is dominated by Y-0 (Figure 2 (yellow)) and Africa is dominated by Y-E (red), where COVID-19 mortality mainly still appears low even if, for example, different age compositions, life expectancy, different quality of registration, makes a valid comparison difficult [4, 12, 13]. A statistical covariation is merely one of the prerequisites to consider when studying possible causality [48]. The present population-based data also suggest a possible biological gradient, which may give a further argument for extended epidemiological studies trying to support or to refute a possible hypothesis on a causal relationship between certain constituencies on the Y chromosome and more serious outcomes from an infection with SARS-CoV-2-virus.

## Conclusions

We propose for further investigation the observed geographical variations in disease severity and their relation to possible biological effects based on sex chromosome variants. The role of population genetics, especially the Y chromosome, has apparently been underexplored in explaining ethnic and gender differences in COVID-19 outcomes [16, 27]. Recent reviews emphasise the need to include the Y chromosomes in whole-genome analyses [49]. Epidemiological data indicate that variations in Y chromosome haplogroups may be associated with increased COVID-19 mortality. Further research is needed to understand possible biological mechanisms behind observations on covariation and in particular their implications for disease susceptibility. Integrating data from epidemiological and basic biological research could enhance our understanding of Y chromosome behaviour and its impact on health, potentially informing therapeutic approaches for a range of diseases.
